# Quantifying carbon in tree bark: The importance of bark morphology and tree size

**DOI:** 10.1111/2041-210X.13546

**Published:** 2021-01-14

**Authors:** Mathias Neumann, Michael J. Lawes

**Affiliations:** ^1^ Department of Chemistry and Biotechnology Faculty of Science, Engineering and Technology Swinburne University of Technology Hawthorn Vic. Australia; ^2^ Institute of Silviculture University of Natural Resources and Life Sciences Vienna Austria; ^3^ School of Life Sciences University of KwaZulu‐Natal Scottsville South Africa

**Keywords:** *Allocasuarina*, allometry, bark fissure index, bark thickness, bark void ratio, biomass functions, *Callitris*, *Eucalyptus*

## Abstract

1. Bark contributes approximately 20% to the total above‐ground biomass of trees, yet bark is not properly accounted for when estimating carbon sequestered by trees. Current allometric functions estimate tree volume from diameter measured over the bark, and derive bark density and carbon content from estimates for wood. As the bark density of hardwood species is 40%–50% lower than the wood density, but nearly equivalent in conifers, bark carbon is overestimated for most species. The latter is further exacerbated by variation in bark volume with bark surface morphology.
2. Fissured bark volume is overestimated by diameter over bark measurements by up to 40%. The vacant space in fissures can be accounted for by a bark fissure index (BFI). We calculate bark carbon for Australian species from a non‐destructive and effective BFI using bark thickness measured in the field.
3. Bark volume, and in turn bark carbon, scaled inversely with tree size (diameter) so that bark volume comprised 42% of small trees (10 cm diameter at breast height, DBH) but 23% of large trees (50 cm DBH). Our BFI method using a bark thickness gauge (BGM) yielded similar results than using the less time‐efficient contour gauge method (CM) to estimate BFI (bias BGM‐CM −1.3%, non‐significant at *p* = 0.72). Both BGM and CM had an error of <4% compared to digitized BFI from destructive sampled stem disks. An average of 15 bark gauge measurements per tree estimated bark thickness (and inconsequence BFI) for both fissured and unfissured bark with <20% error relative to the exact value.
4. Using the bark gauge method, BFI can be rapidly measured from large numbers of trees needed for estimating bark carbon at the community level and modelling carbon uptake, storage and cycling in woody biomes.

## 1 INTRODUCTION

Bark comprises all tissues outside the vascular cambium. In older trees, the bark is divided into inner and outer bark, or the living phloem and the rhytidome (dead phloem and periderm), respectively (Rosell, 2016). The dead outer bark, sometimes referred to as the cork cambium, is a defining feature of many tree species. Bark thickness varies considerably among species (Jackson et al., 1999; Rosell, 2016) and can represent a substantial share of tree volume or biomass (Chang et al., 2020). Yet, the contribution of bark is seldom accounted for in growth models and allometric functions for estimating volume, biomass and carbon allocation among tree species (Chave et al., 2014; Neumann et al., 2016). Nearly all of these models rely on stem circumference or diameter measured over the bark, even though bark and wood (secondary xylem) have very different density and carbon content (Rosell, 2016; Williams et al., 2007). As carbon represents about half of biomass, and carbon content is related to energy content (Thomas & Martin, 2012), the amount of carbon sequestered by bark is important for modelling changes in atmospheric CO_2_, greenhouse gas assessments, monitoring the effects of climate change and the increasing demand for (bio‐)energy (Chave et al., 2014; Preece et al., 2017). Methods for determining bark decomposition and mass loss have been developed (Chang et al., 2020), but robust methods for measuring bark biomass are lacking.

To include bark in estimates of stem volume, we propose a method of estimating the biomass and carbon contribution of bark from readily available bark gauge data. A simple bark typology distinguishes among smooth, white, fissured and scaly bark (Nicolai, 1986). Many broadleaf tree species have smooth or white bark, particularly as juveniles, such as *Betula*, *Populus* or *Fagus* spp. in temperate–boreal forests (Nicolai, 1986), *Adansonia* spp. native to Africa and introduced to Asia (Kamatou et al., 2011), *Shorea* spp. in SE Asia (Gautam & Devoe, 2006) and many *Eucalyptus* and *Corymbia* species in Australia (Grootemaat et al., 2017; Lawes et al., 2020). Other broadleaf trees have fissured bark (e.g. *Quercus* spp. or *Fraxinus* spp.; Costa et al., 2003; Whitmore, 1963) as do many coniferous tree species, such as *Pinus* spp. (Keeley, 2012). Conifers may also have scaly bark, for example, *Tsuga*, *Cedrus*, *Picea* and *Abies* spp. (Van Mantgem & Schwartz, 2003). While smooth, white and scaly bark types are compact with few air pockets, the corrugated nature of fissured bark results in vacant space being included in measures of tree diameter taken over the bark. Also, ‘smooth’ bark types can exhibit fissures caused by mechanical injury, fire, disease, lichen or fungi, or as natural product of age (MacFarlane & Luo, 2009). In general, the volume ratio of bark to wood depends on genus, habitat and environmental conditions such as fire type (Adams & Jackson, 1995) or bark moisture (Wesolowski et al., 2014). Bark thickness varies considerably among biomes and is relatively thicker in fire‐prone biomes such as tropical savanna (Dantas & Pausas, 2013; Schafer et al., 2015). In addition, bark thickness increases with tree age while the ratio of bark to wood declines (Jackson et al., 1999; MacFarlane & Luo, 2009). Thus, to estimate the contribution of bark to the carbon pool, it is necessary to measure bark volume relative to tree size and species at the tree community level.

Here we combine a bark fissure index (BFI) derived from bark thickness measurements, bark basic density and carbon content of bark, to estimate bark carbon relative to tree diameter or volume. BFI assesses the extent of bark fissuring from bark thickness measured at several random points around the circumference of the tree. Our index is a variant of a BFI developed to describe the water storage capacity of bark (Ilek & Kucza, 2014), which, in turn, is derived from an index developed by MacFarlane and Luo (2009) to assess habitat suitability for bark‐dwelling organisms. Other attempts to describe bark allometry and BFIs, of various logistic and technical complexity, have been proposed (Adams & Jackson, 1995; Ilek & Kucza, 2014; Van Mantgem & Schwartz, 2003). We apply our easily measured BFI to estimating the biomass contribution of bark from Australian examples of fissured and scaly bark and demonstrate its efficacy for field studies of carbon storage in tree communities.

## 2 MATERIALS AND METHODS

**FIGURE 1:**
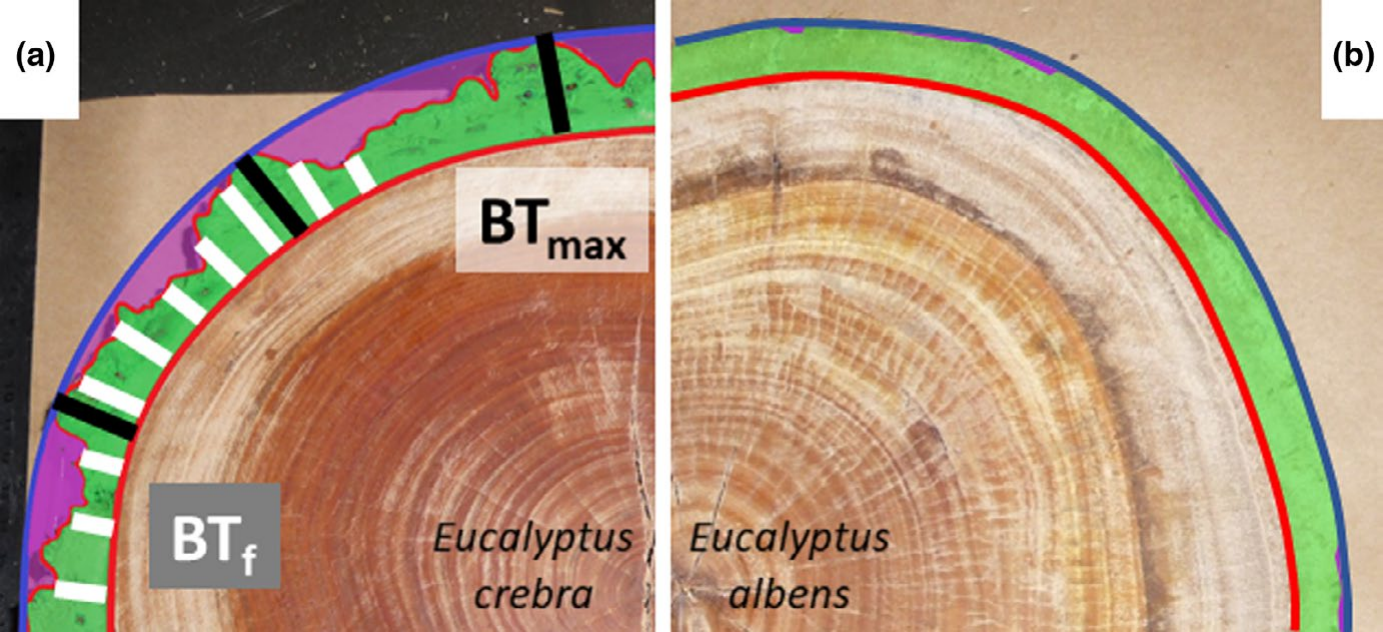
Fissured and scaly bark types of four Australian tree species: *Eucalyptus crebra* (a) and *Eucalyptus albens* (b). Red line is the wood surface, blue line the outer bark perimeter, green shaded area is the inner and outer bark combined, and the purple shaded area is vacant space between bark surface and outer bark perimeter. Black line segments indicate maximum bark thickness, BT_max_ and white segments measurements for fissure‐corrected bark thickness, BT_f_

To test and validate our BFI, we selected four coexisting tree species from semi‐arid dry sclerophyll forests in New South Wales, Australia (Neumann et al., 2020), two eucalypts, one casuarina and one native conifer (Figure 1). *Eucalyptus crebra* F. Muell., the Narrow‐leaved Red Ironbark, has thick bark, that is deeply fissured and is shed irregularly. The bark is persistent to the small branches. In contrast, *Eucalyptus albens* Benth., White box, has sub‐fibrous finely tessellated bark that is shed to reveal the inner smooth white bark. *Allocasuarina luehmannii* (Aiton) L.A.S. Johnson, known as Buloke or Bull‐oak, has the hardest known wood (also called ironwood) and rough‐fibrous and fissured bark. Finally, *Callitris glaucophylla* J. Thomps. & L.A.S. Johnson (synonym *Callitris columellaris* F. Muell.), the White Cypress‐pine, is a native conifer in Australia (Cupressaceae). It has persistent bark that is hard and deeply fissured. The study site is a managed forest located at −32.231°S, 148.949°E (Neumann et al., 2020).

### 2.1 Relative bark area using bark thickness measurements

We secured four stem disks each from all four species in November 2018 from pole‐sized stems in the diameter range 20–30 cm. Disks were harvested from 1.3 m height above‐ground. We partitioned each air‐dried disk into four quadrants and measured bark properties for each quadrant separately. We measured maximum bark thickness, BT_max_, from the wood surface to the outer bark surface at ridges at three points per quadrant and calculated the mean. For smooth bark, we measured bark thickness at 10 points per quadrant and calculated BT_max_ as the mean of the three largest values. The diameter of the stem over bark was measured using a diameter tape, and used to calculate the relative bark thickness (RBT, BT_max_/stem diameter, Equation 1; Midgley & Lawes, 2016) and the relative bark area (RBA, bark area/stem area, Equation 2).

(1) $$\text{RBT}\;\text{(}\%\;\text{cm}/\text{cm)}\;=\;2\;{\text{BT}}_{\text{max}}/(\text{DoB}\;‐\;{\text{2 BT}}_{\text{max}}),$$

(2) $$\begin{array}{cc} \text{RBA (}\%\;{\text{cm}}^{2}/{\text{cm}}^{2})\; & =\;\left({\text{DoB}}^{2}\pi /4\;‐\;{\text{DuB}}^{2}\pi /4\right)/{\text{DoB}}^{2}\pi /4\\ & =\;\left({\text{4 BT}}_{\text{max}}\text{DoB}\;‐\;{\;\text{4 BT}}_{\text{max}}^{2}\right)/{\text{DoB}}^{2}, \end{array}$$

DoB is the diameter over bark (cm), DuB (=DoB − 2 BT_max_) is the diameter under bark (cm), BT_max_ is the maximum bark thickness, measured at ridges for fissured bark (cm). Equation 1 is from Midgley & Lawes, 2016) and Equation 2 is derived by substituting into and simplifying the equation for the area of the bark annulus divided by the area of the stem over bark assuming a circular shape.

Using RBA, we can calculate the area of bark from total stem area, measured by basal area counts or fixed‐size plots. Bark thickness for this purpose is the standard measure of the distance between bark surface and wood surface; in other words, the distance between the projected surface when using a diameter tape or a calliper and the wood surface. This ‘maximum’ bark thickness BT_max_ is also commonly used for RBT, an important plant trait often linked to fire resistance in fire‐prone savannas and woodlands (Lawes et al., 2013; Pausas & Bond, 2020), and should be calculated relative to bole or the wood diameter under bark to avoid autocorrelation (Schafer et al., 2015). RBA can be applied to stem volume estimates if we assume that bark thickness and texture are proportional along the stem to the branch tips. This assumption does not apply to half‐butt eucalypts or tree species whose bark thickness varies significantly along the stem axis (Lawes et al., 2020).

### 2.2 Accounting for bark fissures with non‐destructive measurements

To account for the space included in current estimates of fissured bark volume (delineated by the wood surface and the hypothetical outer bark surface; Figure 1), we defined a ‘bark fissure index’ (BFI), which is 1 for smooth bark without any fissures, to allow for simpler calibration of bark area. Our BFI is different from the BFI defined by MacFarlane and Luo (2009) with unit centimetres or the ‘bark void factor' of Miles and Smith (2009), where BFI = 0 denotes no fissures.

Our emphasis was on a rapid method for deriving BFI using bark gauge measurements that is suited to ecological studies of carbon storage. We validated our method against (a) a variant of the ‘contour method’ (CM) proposed by Adams and Jackson (1995) and (b) digitized estimates of bark area (DM) from stem disks. For (a), we measured the bark profile using a contour gauge (about 20 metal pins/cm) and the bark thickness at three points (start, centre and end of profile). We then digitized the bark profile and reconstructed under and over bark contours using Adobe Photoshop. We used the same procedure as for method ‘CM2’ by Adams and Jackson (1995), apart from measuring bark thickness also in the centre of the bark profile, in addition to start and end. We calculated BFI_CM_ by dividing the area of solid bark by the area of the idealized annulus of the bark area (measured with a diameter tape). BFI_CM_ was measured for the four quadrants of each disk in our study. Similarly, for (b) we calculated BFI_DM_ by dividing digitized solid bark by bark annulus area.

For rapid measurement of BFI in the field, we used multiple bark thickness measurements using a bark gauge around the circumference at a predetermined height (usually at breast height or 1.3 m) on the stem. The mean of these measurements is taken to be the bark thickness corrected for fissures (BT_f_) with the same assumption of bole shape as Adams and Jackson (1995), that the inner limit of the bark or bole shape is not similarly irregular. Accounting for variation in bark thickness in this way, BT_f_ can be used to estimate BFI (Equation 3). The measurements can be taken randomly or systematically (e.g. every 1 cm or at predetermined compass bearings), but for unbiased results it is imperative to measure bark thickness only between bark surface and wood and not from the outer projected bark surface (i.e. not solely from ridges, which measures BT_max_, but within fissures too). This may require a narrow bark gauge that can be inserted into fissures. We measured BT_f_ at 40 points per stem disk for all four tree species with or without clear fissures (Figure 1).

(3) $$\text{BFI}\;=\;{\text{BT}}_{f}/{\text{BT}}_{\text{max}}.$$

We then used the coefficient of variation (CV) from the 40 bark thickness measurements to estimate the error of only five bark thickness measurements—a commonly suggested sample size (*n*) per tree (Pérez‐Harguindeguy et al., 2013; Stängle et al., 2015). Error is ((CV *t*)^2^/*n*)^0.5^, with *t* the 95% quantile of the two‐tailed *t* distribution with *n* − 1 degrees of freedoms (Stängle et al., 2015). We repeated the error calculations for *n* equal 10, 15, 20 and 30.

Using the 40 bark thickness measurements, we calculated BFI for each of the four disk quadrants from 10 measurements each, respectively, using Equation 3. This ensured the same number of measurements per stem disk for comparison with BFI_CM_ (*n* = 4).

### 2.3 Calculating bark carbon with over bark tree measurements

With RBA and BFI, we can estimate the share of solid bark and can calculate bark carbon (Bark C) from more readily available basal area (BA):

(4) Bark C=BA·H·FF·RBA·bark density·carbon fraction·BFI.

BA is basal area (m^2^), H the tree height (m), FF the form factor (ratio of basal area and stem volume over bark), bark density has unit kg/m^3^ and carbon fraction (kgC/kg or %C), thus bark C has unit kgC. Multiplying by BFI accounts for an irregular bark surface in fissured bark types. We measured bark density using the displacement method after saturating bark samples, and carbon fraction by dry combustion (Neumann et al., 2020).

Equation 4 can be modified to use stem volume (V) over bark (m^3^), BM stem biomass (kg) or tree carbon (kgC) by substituting the terms BA · tree height · FF (Equations (4), (5), (6)). Equations (5), (6), (7) assume that density and carbon content of bark are the same as density and carbon content of wood and will result in overestimates of bark carbon, as bark density is usually less than wood density (Miles & Smith, 2009; Neumann et al., 2020). Conceptually, Equation 3 can also be used to convert fluxes, such as net primary production or biomass increment rates (Landsberg et al., 2003).

(5) BarkC=V·RBA·barkdensity·carbonfraction·BFI,

(6) BarkC=BM·RBA·carbonfraction·BFI,

(7) BarkC=Tree C·RBA·BFI.

We analysed using Equation 5 the effect of tree size on total bark carbon of 1 m^3^ wood for four hypothetical smooth‐barked trees (not measured) with 2, 10, 30 and 50 cm diameter over bark at breast height (DBH), correcting for BFI using values from *E. albens*. RBA was corrected using bark thickness calculated using an allometric bark thickness model for eucalypts (Muhairwe, 2000).

## 3 RESULTS

For species with fissured bark, a substantial share of RBA is air‐filled (Table 1a). While RBA was largest for the two eucalypts, there was more carbon in the bark of *C. glaucophylla* and *E. crebra* due to greater bark density (Table 1b). The bark fissure index BFI ranged from 63% to 95% for the four tree species ($\bar{x}$ = 82%, *n* = 16). There was no significant difference between estimates of BFI_BGM_ and BFI_CM_ (*p* = 0.72, two‐tailed *t*‐test) and the difference ranged from −9% to +11%, with bark gauge estimates of BFI on average 1.3% lower than the contour method ($\bar{x}$ = −1.3%; BFI_BGM_ − BFI_CM_, *n* = 16). Both methods captured the lower BFI of fissured *E. crebra* (~67%, one‐third of bark volume is air‐filled) and higher BFI+ 90% for smooth‐barked *E. albens*. BFI estimated from digitized bark area (BFI_DM_) was assumed to be most accurate and used as a baseline for absolute bark area. The BFI_BGM_ estimates were within −5% to +15% of the ‘true’ BFI_DM_ values ($\bar{x}$ = +4.0%; BFI_BGM_ – BFI_DM_). Digitized estimates showed similarly low BFI for fissured *E. crebra* and higher BFI for smooth bark types, but were consistently than those from BGM and CM.

**TABLE 1:** (a) Relative bark area (Equation 2) from estimated bark basal area for two Eucalypt species, one native conifer and one Casuarina. BT_max_ is the maximum bark thickness and BT_f_ accounts for fissures. (b) Bark carbon per m^3^ using carbon density and bark fissure index, BFI (Equation 5). We compare BFI using the bark gauge method introduced here (BFI_BGM_; $\bar{x}$ ± *SD*, *n* = 4), with the contour method (BFI_CM_; Adams & Jackson, 1995) and digitizing stem disks (BFI_DM_). Carbon density is specific bark density multiplied by bark carbon fraction from Neumann et al. (2020) and bark carbon is calculated with Equation 5 and BFI_CM_. (c) The effect of tree size on bark carbon for four hypothetical unfissured, smooth‐barked *Eucalyptus albens* with DBH 2, 10, 30 and 50 cm

(a) Relative bark area					
Species	Diameter over bark (cm)	BT_max_ (cm)	BT_f_ (cm)	RBT	RBA
*Eucalyptus crebra*	28.3	3.1	2.1	28.1%	39.0%
*Eucalyptus albens*	20.3	1.0	0.9	11.0%	18.8%
*Callitris glaucophylla*	25.4	1.1	1.0	9.5%	16.6%
*Allocasuarina luehmannii*	28.0	0.9	0.8	6.9%	12.4%

(b) Bark carbon for 1 m^3^ volume over bark					
	BFI_BGM_ = BT_f_/BT_max_	BFI_CM_	BFI_DM_	Carbon density (kgC/m^3^)	Bark carbon (kgC)
*Eucalyptus crebra*	66.2 ± 6.0%	67.5 ± 3.5%	74%	240	63
*Eucalyptus albens*	91.3 ± 2.7%	92.1 ± 6.0%	90%	195	34
*Callitris glaucophylla*	85.1 ± 2.1%	86.0 ± 1.9%	99%	330	47
*Allocasuarina luehmannii*	83.8 ± 3.5%	86.1 ± 5.0%	80%	253	27

(c) Effect of tree size on relative bark area and bark carbon of 1 m^3^ volume over bark				
	Seedling	Young tree	Middle‐sized tree	Large tree
DBH (cm)	2	10	30	50
2 · BT_max_ (cm)	0.8	2.8	5.3	6.2
RBA · BFI (%)	64%	48%	32%	23%
Bark carbon (kgC)	115	87	58	42

The digitizing method (DM) was the least time‐efficient method, with ~20 min to fell the tree and remove the stem disk, 15 min to prepare the disk and 20 min to digitize bark area and complete the calculations. The contour method (CM) took ~30 min per quadrant, including measuring the contour and bark thickness at three locations, digitizing and calculating. The time needed for bark gauge method (BGM) varied with the number of bark thickness measurements and 10 measurements (used for the comparison with BFI_CM_) and calculating BFI with Equation 3 took about 5 min.

We examined the effect of tree size on the bark carbon of a constant wood volume (Table 1c). Bark thickness increased with tree diameter (i.e., age), from 0.8 cm for a 2 cm seedling, to 6.2 cm for a tree with 50 cm DBH, but RBT (Equation 1) decreased from 67% to 14%, respectively. Assuming BFI does not change with age, bark comprises 64% (RBA multiplied by BFI) of the bark area of a sapling with a 2 cm diameter over bark, whereas for a large tree with 50 cm DBH this share is about 20%. The relative bark area and the bark carbon of 1 m^3^ tree volume is thus inversely related to bark thickness and decreases with tree size. At the stand level 100 m^3^ volume measured over bark from 10 cm DBH trees contains 8,700 kgC, while the same volume from 50 cm DBH trees contains 4,200 kgC. Bark proportions decline from 63% for 2‐cm seedlings to 23% of 50‐cm trees, as relative bark thickness decreases with increasing bole or wood diameter (Table 1c).

Finally, we examined the effect of increasing the number of bark gauge measurements on BT_f_ needed to derive BFI_CM_ (Equation 3). We measured bark thickness 40 times for each disk, while five per tree are commonly recommended (Pérez‐Harguindeguy et al., 2013). Five measurements for the smooth‐scaly barked *E. albens* and *C. glaucophylla* yielded a BT_f_ error of approximately ±20% or ±0.03 cm, compared to the exact BT_f_ value (Table 2). For the fissured species, *E. crebra* and *A. luehmannii*, at least 15 measurements were required to achieved an equivalent BT_f_ error of 20% (Table 2).

**TABLE 2:** Estimate error of fissure‐corrected bark thickness, BT_f_, for increasing number of bark thickness measurements. Mean, standard deviation and coefficient of variation (CV) were calculated using 40 samples. We provide error relative to CV in % and in absolute terms (cm) for increasing sample number *n* from 5 to 30

Bark thickness fissure‐corrected	*Eucalyptus crebra*	*Eucalyptus albens*	*Callitris glaucophylla*	*Allocasuarina luehmannii*
Mean BT_f_ (cm)	2.08	0.94	1.00	0.77
*SD* (cm)	0.74	0.15	0.15	0.29
CV (%)	35.75	15.77	15.35	37.99
Number samples	40	40	40	40
Error (% or cm) for *n* = 5	44% 0.33 cm	20% 0.03 cm	19% 0.03 cm	47% 0.14 cm
Error (% or cm) for *n* = 10	26% 0.19 cm	11% 0.02 cm	11% 0.02 cm	27% 0.08 cm
Error (% or cm) for *n* = 15	20% 0.15 cm	9% 0.01 cm	9% 0.01 cm	21% 0.06 cm
Error (% or cm) for *n* = 20	17% 0.12 cm	7% 0.01 cm	7% 0.01 cm	18% 0.05 cm
Error (% or cm) for *n* = 30	13% 0.10 cm	6% 0.01 cm	6% 0.01 cm	14% 0.04 cm

## 4 DISCUSSION

Here we introduce a method for calculating the proportional contribution of bark to whole tree carbon. Our bark gauge method (BGM) accounts for fissured bark thickness using a bark fissure index (BFI) and is verified against two alternative methods: (a) digitizing bark area (DM)—the assumed most accurate method and (b) the contour method (CM) after Adams and Jackson (1995). The BGM relies solely on bark thickness measurements and takes 10%–20% of time needed for the contour method. BGM and CM provide similar estimates of BFI and bark area, but the CM is more cumbersome and less applicable to field studies than the BGM. The DM, on the other hand, requires stem disks and is thus destructive. Furthermore, bark fissures can be obscured in cross‐sectional view, particularly if fissures are at an acute angle to the stem axis. For example, clearly fissured *Callitris glaucophylla* had a BFI close to 1 for DM, but a BFI of <0.9 for either the BGM or CM. An accurate BFI method has to capture the often fine‐scaled bark structure along the stem circumference and both the BGM and CM accomplish this by providing comparable results, with BGM being more efficient for field studies.

We show how RBA can be combined with BFI, specific basic density and carbon fraction to determine bark carbon. Applications of our method include estimating bark carbon from over bark measurements (e.g. Hudak et al., 2014; Lang et al., 2016), field measurements of bark decay (Chang et al., 2020) and harvest assessments or estimating bark volume of standing dead trees (Chave et al., 2014; Neumann et al., 2020; Woldendorp & Keenan, 2005), and can be combined with a broad compilation of bark thickness models (Jackson et al., 1999). Our method can be applied to any tree species or woody biome. To facilitate this, we discuss the importance of bark density, bark morphology and tree size to estimating bark carbon.

### 4.1 Importance of bark density

Bark density is important for determining bark carbon (Table 1), as well as for fire resistance and flammability (Grootemaat et al., 2017; Lawes et al., 2011; Wesolowski et al., 2014). Bark density can be calculated using the specific gravity method (Pérez‐Harguindeguy et al., 2013) or, as is often the case in boreal or temperate regions, by assuming that bark and wood density of bark is the same as wood density. The latter assumption can result in large errors in hardwoods from tropical regions, such as the eucalypt species in this study (cf. Ilic et al., 2000; Neumann et al., 2020). Quantifying the density of outer flaky and loose bark (sensu Rosell, 2016) is challenging and most bark density values represent predominantly consolidated inner bark.

There is no database of bark density for Australian tree species. Indicative bark densities are drawn largely from a North American study (Miles & Smith, 2009). The ratio of wood to bark density (both oven‐dry and relative to green‐wet volume) is 1.013 ± 0.247 (ranging from 0.6 to 2.28). In North American tree species, wood is on average 1.3% denser than bark. The assumption for North America that bark density is equal to wood density does not hold at the regional level where the error can be as large as 200% (Miles & Smith, 2009). We expect similar variation in Australia. Native species in the Myrtaceae dominate woody biomes in Australia and in general have a lower bark than wood density. In plantation‐grown *Eucalyptus globulus* in Portugal, bark density is less than wood density by 17% (bark 473 kg/m^3^, wood 567 kg/m^3^; Miranda & Pereira, 2016). In Australia, bark density is 50% lower for *E. crebra* (bark 439 kg/m^3^, wood 870 kg/m^3^), and 44% lower for *E. albens* (bark 464 kg/m^3^, wood 827 kg/m^3^; Neumann et al., 2020). This pattern also applies to non‐eucalypts such as *Allocasuarina luehmannii*, a species known for its hard and dense wood (bark 450 kg/m^3^, wood 985 kg/m^3^). In contrast, the bark density of the native conifer *Callitris glaucophylla* is similar to wood density (bark 608 kg/m^3^, wood 624 kg/m^3^).

Using our BFI in combination with estimates of bark density, existing biomass functions can be used to calculate bark carbon (e.g. Chave et al., 2014). These functions estimate above‐ground biomass from diameter, height and basic density (i.e. assumed constant for bark and wood). Bark carbon calculations using our Equations (2), (3), (4) for two trees with differing bark morphology (see following section) show that more than one‐third (36%) of above‐ground biomass of the smooth‐barked tree is bark, but for the fissured tree it is 24% multiplying RBA and BFI (Box 1); both examples stress that bark cannot be ignored in biomass functions and carbon estimates. Calculating bark C for a fissured tree based on BT_max_ and thus not considering bark fissures (assuming BFI of 1, *cf*. smooth‐barked tree) through BFI using one of the above methods (BGM, CM or DM) would overestimate bark C by 49%.

BOX 1 **Example of calculations of carbon (C) in bark using current allometric functions for two trees with the same maximum bark thickness (BT_max_) but contrasting bark morphology (BT_f_ fissure‐corrected bark thickness). DoB is diameter over bark, H tree height, AG BM above‐ground biomass, RBA Relative bark area, BFI bark fissure index**

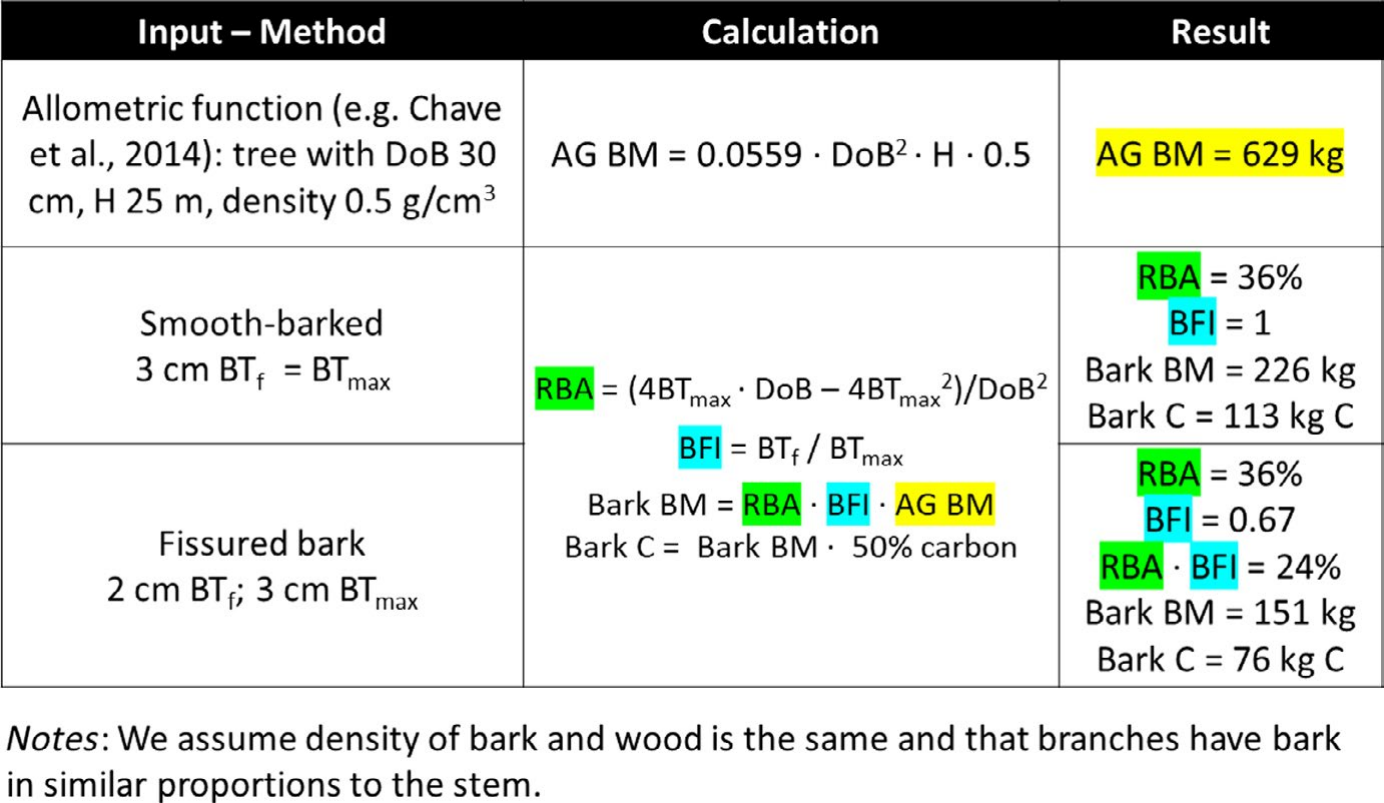

### 4.2 Bark surface morphology matters

We have shown that not accounting for bark fissures results in an overestimation of bark volume (and in turn, its carbon and energy equivalents) by >40% (Table 1) for fissured bark types like ironbark eucalypts such as *Eucalyptus crebra*. We expect similar results for fissured *Quercus* or *Populus* spp. Even rough field‐based BFI estimates provide more realistic numbers for bark volume than ignoring this common feature of many tree species worldwide (MacFarlane & Luo, 2009). Measuring fissured bark systematically at the ridges and in between (e.g. Pérez‐Harguindeguy et al., 2013) assumes regular triangular‐shaped bark fissures, corresponding to a BFI of 0.5—an assumption not supported by our data (Figure 1, Table 1). A more realistic BFI is achieved if bark thickness is measured at equally spaced intervals or randomly around the circumference of the tree. For tree species with homogeneous bark (coefficient of variation of ~15% of mean BT), such as smooth‐scaly barked *Eucalyptus albens* and *Callitris glaucophylla*, five bark thickness measurements are sufficient to obtain fissure‐corrected bark thickness. Our method permits evidence‐based decisions on sampling density that can be easily expanded to other tree species. Fissured and heterogeneous bark require at least 15 measurements to obtain bark thickness estimates with <20% error or approximately 0.2 mm (Table 2). Bark gauges overestimate real bark thickness by about 0.5 mm or even higher (Stängle et al., 2015) and thus improving the accuracy of the bark gauge tool would be useful.

Bark surface morphology varies considerably among tree species. In the ‘half‐butt’ Eucalypts, for example *Eucalyptus pilularis or E. miniata*, rough fibrous and thick outer bark at the base, gives way to thin white outer bark on the branches (Lawes et al., 2020). *Pinus sylvestris*, common in temperate and boreal forests, has a similarly variable bark morphology, with thick, fissured bark at the stem base and often thin red bark on the upper stem and branches (San‐Miguel‐Ayanz et al., 2016). This ‘dual bark’ feature, as well as the varying bark thickness between tree species, is the result of growth and mortality of bark layers along the stem and branches. Some trees accumulate outer bark, potentially as protection against mechanical damage, herbivory and or fire (Pérez‐Harguindeguy et al., 2013; Van Mantgem & Schwartz, 2003), while others shed their outer bark regularly so that only the most recently produced bark layers are present. Reasons for bark shedding include protection against herbivory, reducing the prevalence of flammable bark slabs or nutrient cycling (Grootemaat et al., 2017; Wesolowski et al., 2014). Bark morphology of trees varies greatly and is a reflection of the adaptive response to persistent environmental conditions (Pérez‐Harguindeguy et al., 2013). Further quantitative data on bark morphology (such as BFI and its variation within the species, between species and habitats) will provide novel insights of the response of forested biomes to climate change.

### 4.3 Larger trees have less bark relative to tree size

The large size of trees, their longevity and changes in tree allometry with increasing size and age are central considerations for estimating tree growth, ecology and population dynamics (Chave et al., 2014; Landsberg et al., 2003). Bark—like any other component of forest ecosystems—is subject to growth and mortality. While bark thickness increases with diameter/age, relative bark thickness as well as relative bark area RBA decreases (Table 1c). The magnitude of this decrease will vary depending on bark allometry (Jackson et al., 1999) and whether BFI changes with tree size (MacFarlane & Luo, 2009). We expect that the relative decrease in bark share in tree volume/biomass/carbon is a universal feature of trees, since bark is shed, while wood is persistent, until internal decay progresses (Roxburgh et al., 2006).

Repeated assessments of dead trees permit tracking of the decay of bark and rates of wood versus bark decomposition (sensu Chang et al., 2020). Bark dynamics, on the other hand, are challenging to quantify (Pook et al., 1997). The annual rates of bark loss (‘sloughing’) and how tree size and or climate conditions alter bark shedding rates are relevant to both carbon assessments and determining fire risk. Radial growth of bark can be determined from measured bark thickness and tree age (Midgley, 2019) or measuring tangential strain and radial growth of wood (Whitmore, 1963). There are few studies of radial bark phloem growth (Clair et al., 2019; Midgley, 2019). Shrubby Protaeceae of South Africa (*Protea nitida*, *P. repens*, *P. laurifolia*, *P lepidocarpodendron*, *Leucospermum concarpodendron*) had annual bark growth rates between 0.2 and 1 mm/year (Midgley, 2019). Whitmore (1963) reports annual growth of phloem of *Quercus robur* and *Castanea sativa* between 0.4 and 1.6 mm/year and for *Fagus sylvatica* of <0.1 mm/year. This suggests that some tree species have a very slow turnover of phloem and it may take more than 50 years for complete renewal of phloem tissue, for example in smooth‐barked *Fagus sylvatica*. For fissured and scaly tree species (*Quercus robur* and *Castanea sativa*), average phloem turnover is 3–15 years (Whitmore, 1963). Tropical trees also have large variation in annual phloem growth, ranging from 0.7 to 24.5 mm/year (Whitmore, 1963). We note that phloem is just one part of total bark thickness. Radial growth of Cork oak *Quercus suber* bark ranges from 2.8 to 3.3 mm/year (Costa et al., 2003).

In conclusion, our method can help quantify bark for new allometric biomass or volume functions for both stem and branches of tree species with fissured bark. Including a BFI in existing models will significantly improve forest resource assessments, as well as carbon flux models, such as global vegetation models or those used for flux towers. Our method of estimating bark thickness and the BFI is non‐destructive, relatively rapid and as accurate as existing but less efficient methods. Regional differences in bark thickness due to tree size and or species, their bark morphology, the error associated with required field measurements, can now be accounted for more easily in calculating bark share of tree biomass and carbon.

## AUTHORS' CONTRIBUTIONS

M.N. and M.J.L. conceived the ideas and designed the methodology; M.N. collected and analysed the data and wrote the first draft manuscript; M.N. and M.J.L. contributed critically to the drafts and gave final approval for publication.

### Peer Review

The peer review history for this article is available at https://publons.com/publon/10.1111/2041‐210X.13546.

## Data Availability

The data and images used for the analysis are available over https://doi.org/10.6084/m9.figshare.13417382 (Neumann & Lawes, 2020).
